# Factors contributing to farm-level productivity and household income generation in coastal Bangladesh’s rice-based farming systems

**DOI:** 10.1371/journal.pone.0256694

**Published:** 2021-09-10

**Authors:** Shah-Al Emran, Timothy J. Krupnik, Sreejith Aravindakshan, Virender Kumar, Cameron M. Pittelkow

**Affiliations:** 1 Department of Crop Sciences, University of Illinois at Urbana-Champaign, Urbana, Illinois, United States of America; 2 Sustainable Impact Platform, International Rice Research Institute (IRRI), Los Baños, Laguna, Philippines; 3 International Maize and Wheat Improvement Center (CIMMYT), Sustainable Intensification Program, Dhaka, Bangladesh; 4 Farming Systems Ecology, Wageningen University and Research Centre, Wageningen, The Netherlands; 5 Department of Plant Sciences, University of California, Davis, California, United States of America; Szechenyi Istvan University: Szechenyi Istvan Egyetem, HUNGARY

## Abstract

Large changes have taken place in smallholder farming systems in South Asia’s coastal areas in recent decades, particularly related to cropping intensity, input availability, climate risks, and off-farm activities. However, few studies have investigated the extent to which these changes have impacted farm-level crop productivity, which is a key driver of food security and poverty in rainfed, low-input, rice-based systems. The objective of this study was to conduct an integrated assessment of variables related to socioeconomic status, farm characteristics, and crop management practices to understand the major factors influencing crop productivity and identify promising leverage points for sustainable development in coastal Bangladesh. Using a panel survey dataset of 32 variables from 502 farm households located within polder (coastal embankment) and outside polder systems during 2005–2015, we employed statistical factor analysis to characterize five independent latent factors named here as *Farming Challenges*, *Economic Status*, *Crop Management Practices*, *Asset Endowment*, *and Farm Characteristics*. The factor *Farming Challenges* explained the most variation among households (31%), with decreases observed over time, specifically households located outside polders. Individual variables contributing to this factor included perceived cyclone severity, household distance to main roads and input-output markets, cropping intensity, and access to extension services. The most important factors for increasing crop productivity on a household and per unit area basis were *Asset Endowment* and *Crop Management Practices*, respectively. The former highlights the need for increasing gross cropped area, which can be achieved through greater cropping intensity, while the latter was associated with increased fertilizer, labor, and pesticide input use. Despite the importance of these factors, household poverty trajectory maps showed that changes in off-farm income had played the strongest role in improving livelihoods in this coastal area. This study can help inform development efforts and policies for boosting farm-level crop productivity, specifically through agricultural intensification (higher cropping intensity combined with appropriate and efficient use of inputs) and expanding opportunities for off-farm income as key pathways to bring smallholder households out of poverty.

## Introduction

Approximately 475 million smallholder farmers globally are estimated to produce 80% of all food consumed [1, 2]. Yet, smallholder farmers face enormous challenges to achieve the first two Sustainable Development Goals (SDGs), food security and no poverty, particularly in Asia [3]. Smallholder systems are highly complex and heterogeneous, making it difficult to understand the major challenges farmers face and how constraints at different scales limit progress towards improved livelihoods. A range of socioeconomic and biophysical constraints are present not only at the farm-level (e.g. soil fertility, farm location, and access to capital, labor, inputs, and markets), but also more broadly due to increasing threats from climate change and extreme weather events [4–6]. Previous research has often focused on individual dimensions of smallholder systems, such as investigating disciplinary socioeconomic challenges [7], biophysical constraints [8, 9], or asset limitations such as land, water, and labor availability [10–12]. However, to design more effective agricultural development and extension policies, there is growing recognition that integrated approaches are necessary to understand how a range of on- and off-farm forces contribute to food security and poverty outcomes.

Bangladesh is a country in South Asia with extremely high rural population density (~1000 people km^-2^) and pressing development concerns, particularly in coastal areas due to risks of sea-level rise and severe tropical cyclones from the Bay of Bengal [13]. The majority of coastal agriculture consists of smallholder, low input, rainfed, rice-based systems characterized by low productivity, high labor requirements, small landholdings with increasing fragmentation, frequent flooding during monsoon season, irrigation water limitation, and salinity during *rabi* season, which threatens crop productivity and limits farmers’ options for growing crops other than rice [14–17]. Previous research has shown that important factors influencing food security include inundation land type, crop management practices, flooding, cyclones, soil salinity, irrigation access, household income, household size, and access to markets [4, 18, 19]. However, the relative influence of these factors on smallholder livelihoods remains uncertain because most studies are strongly disciplinary, which, compared to more integrated and interdisciplinary approaches, may fail to account for key drivers and nuances that affect food security. Moreover, relatively rapid changes have taken place in rural infrastructure, farm size, cropping intensity, mechanization, and off-farm economic opportunities in recent decades [15, 20]. From a food security and income perspective, some of these changes represent challenges and other opportunities. Yet few studies have explored how whole farming systems have responded to these changing circumstances and whether the net impacts resulting from change have been positive or negative in terms of crop productivity and poverty reduction.

The goals of smallholders in this region are generally to reduce risks, generate income, and achieve food security. However, they face numerous constraints related to labor availability, access to inputs, poor infrastructure, access to credit and extension services, socioeconomic instability, and low soil fertility levels, among others [14, 20]. Farms in remote locations tend to lack adequate access to roads and transportation; this increases transaction costs and hampers agricultural and economic growth [21, 22]. While efficient use of fertilizers, pesticides, and human labor can increase yields [23, 24], many inputs may not be readily available in markets. In addition, rural agricultural labor availability is decreasing, with workers and youth increasingly interested in non-agricultural professions providing greater income [14]. Many smallholders also lack regular access to quality extension services [25]. Research has documented the importance of low-risk financial credit for enhancing technology adoption and crop productivity [26, 27], though gaining access to credit depends on different factors. Depending on the lender, farm income, assets, age of household head, and household size tend to be important [28].

At the same time, Bangladesh has more than doubled its per capita gross domestic product (GDP) in recent decades, and trends in urbanization are providing new opportunities for off-farm income [29]. When sent as remittances, off-farm activities can bolster farm income, encourage new investments, and build the capacity of smallholders to overcome economic challenges [30]. In southern Bangladesh, it has been estimated that 77% of farmers are unable to obtain a viable livelihood through farming alone [31]. Farm income, monthly savings, crop price, market conditions, and access to inputs and water all impact the vulnerability of smallholder livelihoods [6]. In addition, increasing soil salinity caused by oceanic water intrusion can decrease crop yields and revenue, leading farmers to pursue off-farm activities [32]. Recent work on smallholder systems in Sub-Saharan Africa highlighted the importance of off-farm income and market access for poverty targeting and households achieving sufficient food availability [33]. As a result, these authors concluded that policies should focus on diversifying employment sources, not just agricultural production. Assessing the relative proportion of income derived from crop production compared to off-farm activities in Southern Bangladesh could provide insights into the poverty trajectory of households over time and the role of off-farm income in improving livelihoods.

Policy development to meet the SDGs must balance the needs of targeting specific issues while also reflecting the complexity of smallholder systems. For example, off-farm income may help alleviate economic concerns, but this should not compromise efforts to achieve food security and reduce risks in rural communities through the integration of income sources to both produce and appropriately purchase food. In this context, identifying the factors supporting higher cropping intensity is critical to inform policy and development initiatives concerned with boosting farm-level productivity. This region of Bangladesh has a tropical climate with three cropping seasons (the winter ‘*rabi*’, spring ‘*kharif*-1’, and monsoon summer ‘*kharif*-2’ seasons), providing an opportunity to grow multiple crops on the same land annually. Increased cropping intensity could help address food security concerns because access to new arable land is negligible and existing farmland is declining due to high population pressure [34, 35]. In the north of the country, Bangladesh has increased mean cropping intensity to around 190%, mostly by expanding groundwater irrigation, while coastal areas remain at 150% [31, 36]. Importantly, while surface water supplies could provide irrigation to produce dry season crops in coastal Bangladesh [37], arable land often remains fallow or planted to non-irrigated crops during this season [16, 17]. This fact indicates the need for research to identify the existing challenges and opportunities to overcome the comparatively lower cropping intensity in the coastal regions.

The present study focuses on smallholder rice-based cropping systems in the low-lying coastal zone of Southern Bangladesh. Many farmers in these systems fall below the international poverty line [38] and are exposed to risks, including food insecurity, extreme weather events, soil salinity, tidal flooding, and sea-level rise [38]. Our overarching goal was to characterize farming systems based on an integrated analysis of socioeconomic variables, farm characteristics, and crop management practices to identify promising leverage points for achieving the SDGs through improved agricultural productivity. The specific objectives were to 1) develop factors representing key differences in farm households using statistical factor analysis, 2) evaluate changes in these factors over a decade, 3) assess how these factors affected total farm-level crop productivity, and 4) map farm household poverty trajectories over the study period to identify the key drivers of economic improvement and provide evidence-based policy advice.

## Materials and methods

### Study area

**Fig 1:**
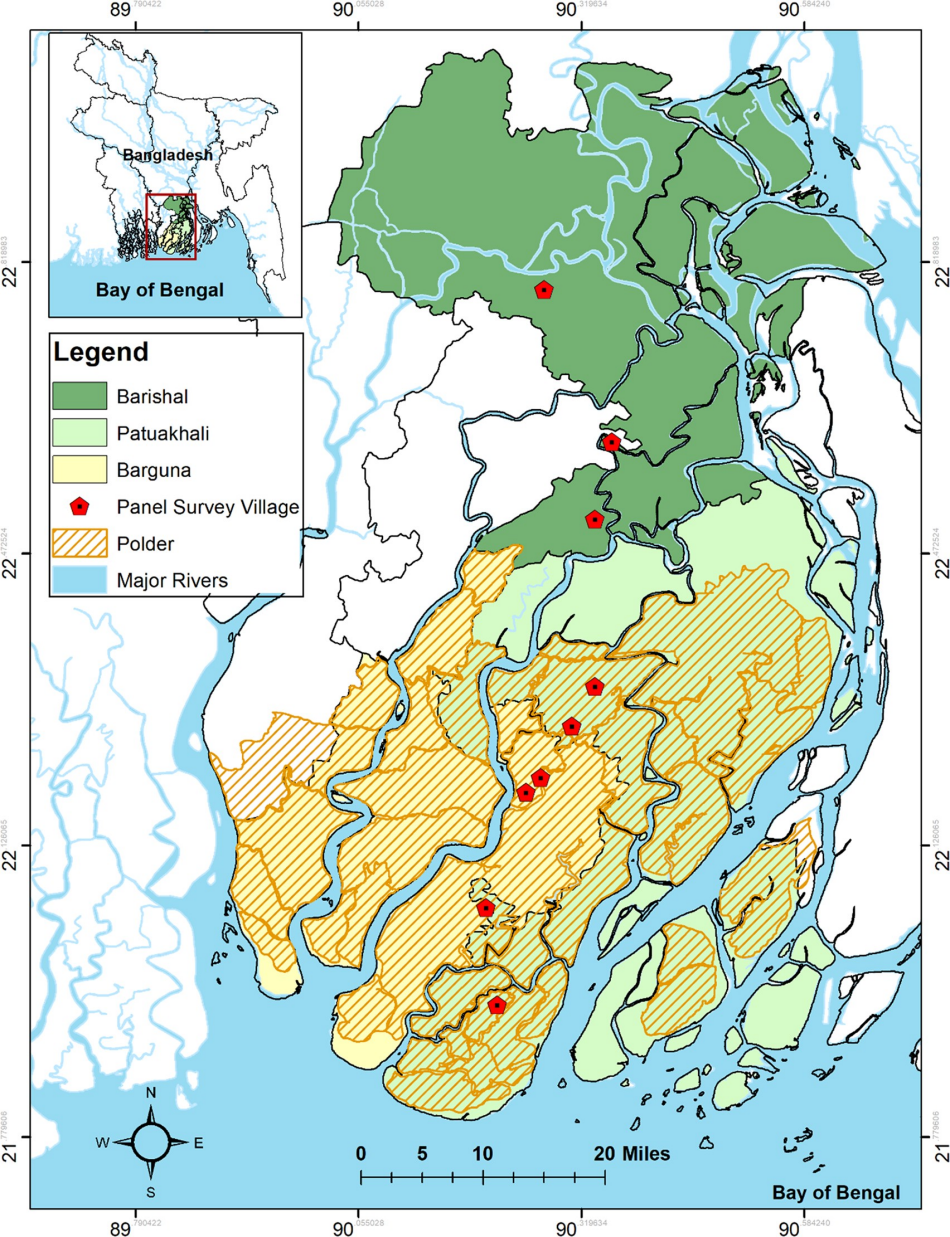
Map of the study area in south-central coastal Bangladesh. The districts covered by surveys are displaying salinity differences as well as the systems of polders.

The study area is the south-central coastal zone of Bangladesh (Fig 1) within the active siltation zone of the Ganges estuary, consisting of many rives and tidal canals in Patuakhali, Barguna, and Barishal districts. These interconnected rivers and canals are responsible for intermittent tidal flooding, land erosion, and siltation during the summer monsoon *kharif*-2 season, which is the most suitable for growing rainfed crops. However, windows in time to cultivate fallow lands by tapping freshwater canals for surface irrigation are also present during the *rabi* season [16]. Depending on proximity to the coastline, farming households in this study are located within polders (near the coast, largely within Patuakhali and Barguna districts in our study area) or outside polders (further north in Barishal). Each area experiences important hydrological conditions, including both oceanic and freshwater water intrusion and cyclones.

Polders are a system of embankments consisting of dykes and sluice gates to provide protection against cyclones, storm surges, fluvio-tidal flooding, oceanic flooding, and saline or brackish water intrusion [39, 40]. Crop failures due to monsoon flooding or saline inundation were common in lower estuarine areas before polder construction started in the 1960s [41, 42]. About 1.3 million ha of land have been protected by constructing more than 123 polders in low elevation coastal zones, containing 5000 km of embankments with 2500 sluice gates or water control structures [43]. Polders are, however, now in a widespread state of disrepair, and the sub-surface intrusion of saltwater contributes to mounting soil salinity concerns, land subsidence, and waterlogging, which are key concerns [17].

The upper estuarine areas (e.g., Barishal district) are outside of polders, meaning they remain vulnerable to tidal flooding during the monsoon season [44]. Research focusing on the saline-prone south-western region of Bangladesh in the Khulna Division has shown that crop productivity, economic performance, and cropping intensity can be improved within polder compared to adjacent outside polder households [42, 45, 46]. However, in regions such as south-central Bangladesh with a reduced risk of salinization and more opportunity for increasing cropping intensity, differences in food production and economic outcomes between polder and non-polder households have been less thoroughly studied.

Both within and outside polders climate is classified as humid subtropical with 1,955–2,100 mm of annual rainfall, falling within the range of optimal precipitation for agricultural livelihoods [47]. The majority soil type is silty clay loam, which is suitable for growing a wide range of crops. Hence, around 70% and 59% households are involved in farming both within and outside of polders, respectively [36]. The months corresponding with the three aforementioned cropping seasons are *kharif*-1 (mid-March to mid-July, pre-monsoon), *kharif*-2 (mid-July to mid-November, monsoon), and *rabi* (mid-November to mid-March, dry winter). Kharif-2 is the major cropping season when rainfed ‘*aman*’ rice is grown by almost all farmers. Hence, rice-based cropping systems are the focus of this study. The *rabi* season is conversely associated with land fallowing or for cultivating irrigated ‘*boro*’ rice, pulses, oilseeds, and in Barishal, vegetables.

Important changes in fertilizer subsidies have occurred during the study period. In 2005, the government of Bangladesh subsidized imported NPK fertilizers for the first time since 1996 [48, 49]. Although subsidies remain to this day, retail prices remain high for rural farmers due to limited dry season cropping and the remoteness of parts of the study area [14]. Moreover, in order to support agricultural development in southern Bangladesh, a policy calling for international donor investment of over USD 7 billion was proposed by the government of Bangladesh for coastal development [38]. Yet despite these policy changes, aid investments and also crop productivity in southern Bangladesh remain the lowest in the country [14].

### Survey data

A household survey was conducted in Barishal, Patuakhali, and Barguna districts of the Barishal division and is publicly available at http://hdl.handle.net/11529/10898. For a detailed description of the methodology and justification of the selected districts, see Aravindakshan et al. (2020) [15]. The dataset contained information on the socioeconomic status, field characteristics, and crop management practices for 502 households collected in five years intervals from 1995 to 2015. For the present analysis, we considered 32 variables using data from 2005, 2010, and 2015 (Table 1). An important reason for this was the adoption of mechanized tillage that occurred in most households from 2005 onwards, representing a significant transition and intensification in crop management. In addition, a new fertilizer policy began in 2005, as described above. In total, data were available for 204 households within polders and 298 households outside of polders. The former households were located in Patuakhali and Barguna districts, the latter in Barishal district.

**Table 1:** Independent variables included in this study^1^.

#	Variable	Description
1	TLO = Total land owned	Land owned by household (ha)
2	FC = Food cropped area	Household annual rice cultivation area (ha)
3	CC = Cash cropped area	Household crop cultivation area except for rice (ha)
4	CRI = Cropping Intensity	$\frac{Gross\;harvested\;area\;(ha)\;{farm}^{-1}{year}^{-1}\;}{Total\;land\;area\;(ha)\;{farm}^{-1}{year}^{-1}}\times 100\;(\%)$
5	MLT = Major Land Type	(1 = Low land, 2 = Medium land, 3 = High land; farmers’ perception on inundation land type)
6	OSF = Overall soil fertility	(1 = Low, 2 = Medium, 3 = High; farmers’ perception)
7	OSS = Overall soil salinity	(0 = No salinity, 1 = Low, 2 = Medium, 3 = High; farmers’ perception)
8	DIC = Distance to irrigation canal	Major crop fields’ distance to the nearest irrigation canal (km)
9	DMR = Distance to main road	Major crop fields’ distance to the main road (km)
10	DIO = Distance to input output markets	Household distance to input/output markets (km)
11	CSI = Cyclone Severity Index	Cyclone severity index was developed by summing up cyclone events’ product and farmers’ perception of an individual cyclone’s severity (on a 0–3 scale) in the observed years.
12	EDU = Education level	Household head education level (years of education)
13	HHS = Household Size	Total people in the household (number)
14	EIC = Experience in cropping	Household cropping experience (number of years)
15	IF = Involvement in farming	Household head involvement in farming (1 = no, 2 = partial, 3 = full)
16	LABA = Labor availability	Availability of labor for farm (1 = No scarcity; 0 = Lack of labor)
17	AC = Access to credit	0 = No access; 1 = Access
18	AE = Access to extension	0 = No access; 1 = Access
19	MTC = Marketed crops	Amount of crop production marketed (%)
20	OI = Off-farm income = Non-crop income	Household annual income other than from crop production (BDT/household)
21	AI = Annual income	Household annual income (BDT/household)
22	FE = Food expense	Household annual food expense (BDT/household)
23	NS = Net savings	Household annual net savings (BDT/household)
24	TCP = Total cost of production	Household cost of crop production (BDT/household)
25	POP = Population	Village level population (number of people)
26	SMT = Share of mechanized tillage	Lands are under machine tillage (% land area)
27	HL = Hired labor	Household hired labor for crop production (psd ha^-1^)
28	FL = Family labor	Household family labor for crop production (psd ha^-1^)
29	TU = Urea	Amount of urea fertilizer used in crop production (kg ha^-1^)
30	TT = TSP	Amount of triple-super-phosphate used in crop production (kg ha^-1^)
31	TM = MoP	Amount of muriate of potash fertilizer used in crop production (kg ha^-1^)
32	PUSE = Pesticide use	Household use of pesticide for crop production (Yes = 1, No = 0)

^1^Variables represent socioeconomic status, field characteristics, and crop management practices in polder and non-polder households of south-central coastal Bangladesh during the study period 2005–2015.

### Computations

Although rice was the predominant crop grown across all households, other crops were also produced. Following standard practice, rice equivalent yield (REY) was estimated to account for total household production, including both rice and non-rice crops. This approach standardizes system performance by including the effect of multiple crops within the rice-based cropping system over time. Both REY and net return were calculated following Eqs 1 and 2 [50]:

$$REY=Y_{x}\times (\frac{P_{x}}{P_{r}})$$ Eq 1

$$\begin{array}{l} Net\;returns\;(Tk\;{ha}^{-1})\\ \;=Gross\;returns\;(BDT\;{ha}^{-1})\\ \;-Cost\;of\;cultivation\;(BDT\;{ha}^{-1}) \end{array}$$ Eq 2

$$Gross\;returns\;Tk\;ha^{-1}=Yield\;t\;ha^{-1}\times Unit\;price\;BDT\;t^{-1}$$ Eq 3

$$\begin{array}{l} Cost\;of\;cultivation\;(BDT\;{ha}^{-1})\\ \;=\sum_{m}^{i}{Inputs}_{i,m}\;(BDT\;{ha}^{-1})+\sum_{j}^{i}{Labour}_{i,j}\;(BDT\;{ha}^{-1})\\ \;+Land\;preparation\;(BDT\;{ha}^{-1})+Irrigation(BDT\;{ha}^{-1}) \end{array}$$ Eq 4

Where *REY* = Rice equivalent yield (t ha^-1^), *Y*_*x*_ = yield of non-Aman rice crop (t ha^-1^), *Px* = price of non-Aman rice crop (BDT t^-1^), and *Pr* = price of *aman* rice (BDT t^-1^). In this calculation, rice price for each household was used to estimate REY, as selling prices differed from household to household. Data for land rent were not available; thus, we did not include this in the cost calculations. Materials, labor, and irrigation operations varied among crops and different households. Different crops and irrigation requirements are also associated with different crops and cropping seasons. We considered both *aman* and *boro* rice as food crops in this study. Crop production was estimated by multiplying REY (t ha^-1^) × cropped area (ha). Household annual crop production and crop productivity were calculated by Eqs 5 and 6:

$$\begin{array}{l} Household\;Annual\;Crop\;Production\\ \;=REY\;(t)\\ \;+Rice\;equivalent\;non\;rice\;crop\;production\;(t) \end{array}$$ Eq 5

$$\begin{array}{l} Household\;Crop\;Productivity\\ \;=\frac{Household\;annual\;crop\;production\;(t)}{Household\;Gross\;Cropped\;Area\;(ha)} \end{array}$$ Eq 6

Prior to data analysis, all economic variables were standardized to 2010 values using the consumer price index (CPI) for Bangladeshi taka, where 1 USD = 69.65 BDT.

Off-farm income (OI) was determined as the amount of income for farm households that did not come from crop production. This was estimated by deducting crop production income or net return from the total reported household annual income. To avoid redundant variables, returns solely from crop production were not used in factor analysis.

To understand poverty trajectories over time, households were categorized into four groups (A, B, C, and D) based on their per capita per day income in CPI adjusted USD equivalent to BDT at the start and end of the study period. Here, A = Per capita income per day ≥ 2 dollar, B = Per capita income per day = 1 to 1.99 dollar, C = Per capita income per day = 0.5 to 0.99 dollar, and D = Per capita income per day < 0.5 dollar. Following the Millennium Development Goals [51], we used the ‘dollar-a-day’ extreme poverty line as a threshold for identifying poor households, meaning households in C and D categories are in extreme poverty. The transition between groups was visualized using a Sankey diagram. The formula for calculating per capita per day income is given below:

$$\begin{array}{l} Per\;capita\;per\;day\;income\;({USD\;day}^{-1}{HHS\;}^{-1})\\ \;=\frac{Household\;annual\;income\;({USD\;year}^{-1})}{Household\;size\times 365\;(day)} \end{array}$$ Eq 7

### Data analysis

Factor analysis was used to quantify relationships among variables with the goal of characterizing households based on key differences among farming systems within and outside of polders. Factor analysis is an efficient tool to quantify the relative impacts of many interacting variables, thereby reducing the complexity of the dataset [52]. Factor analysis has been used to study variability among smallholder crop and dairy farming systems, with a particular focus on technology adoption [53], smallholders’ climate adaptation barriers [54], evaluation of sustainability [55], and evaluation of smallholders’ land-use knowledge [56]. In the broader literature, the documented strength of this method is its ability to objectively group (categorize) multiple variables or develop an integrated index by condensing observed correlations between a large number of variables into a lower number of unobserved or latent factors (variables that are not directly observed but are estimated by factor analysis) [57].

To assess relationships among variables in the survey dataset, a Pearson’s correlation matrix was created using the “GGally” package in R (version 3.5.2). Data were also assessed with regard to normality and heterogeneity of variance assumptions. Exploratory factor analysis was conducted with the 32 independent variables to reduce data complexity and reveal the true dimensionality with as little information loss as possible. Linear relationships between variables were examined before implementing the factor analysis, and variables were correlated enough to support this approach. The sample size (1,506) and sample to variable ratio (47:1) were considered acceptable for factor analysis [52, 58]. The analysis suitability of the respondent data was assessed by the Kaiser-Meyer-Olkin (KMO) measure of sampling adequacy (MSA) test (overall MSA = 0.72). The MSA for each item was >~0.5, indicating they passed the standard for factorability [57]. All variables were first standardized by calculating individual z-scores relative to the overall average value. The extraction method was principal component analysis (PCA) involving a Varimax (orthogonal) rotation. Varimax rotation was selected to identify uncorrelated factors.

Five factors were identified by considering eigenvalues, scree-plot, total variance explained, and interpretability. The scree plot suggested that the number of factors was 5 (i.e., where eigenvalues > 1). In the factor analysis, seven factors showed the sum of squared (SS) loading > 1, explaining 0.58 of cumulative variance. However, using five factors explained 51% of the total variance, which reduced the number of factors to interpret and was considered adequate for this study. The top five variables with a loading score > 0.40 (or when not enough variables met this criterion, the top variables with the highest loading scores) were used to characterize and label the factors for interpretation. Factor scores were calculated using regression, representing the classification of each household on the characterized/categorized factors. To conduct the factor analysis, we used the R packages “psych”, “psychTools”, and “GPArotation”.

Multi-level mixed-effects analysis of variance was conducted to examine the effects of the year (2005, 2010, 2015), embankment status (households within vs. outside of polders), and their interaction for each latent factor. We performed the analysis using the “lme4” package (lmer function) in R, incorporating both random and fixed effects through a series of iterative model testing to improve performance. The model which yielded the lowest Akaike Information Criterion (AIC) score compared to other candidate structures was retained. In the best fit model, the terms year, embankment status, and their interaction were classified as random, fixed, and random, respectively, with household nested within embankment status.

Once the five factors were established, we used them as predictor variables to determine their relative importance on household cropping system productivity (across location and years). For this step, we trained a random forest (RF) model considering two versions of productivity as a response variable, both described above. First, productivity was evaluated on an annual basis, which represents the total crop production output of a household considering REY and all land area under cultivation (Eq 5). Second, productivity was evaluated on a per unit area basis, which represents household annual crop production (REY) per total cropped area (ha) (Eq 6). Each model was trained with 1,000 trees using the “randomForest” package in R [59]. Variable importance plots were created using the “varImpPlot” command. Once the top three most important factors explaining variance were identified, relationships of these predictor variables with REY were displayed using linear regression models. The “plyr”, “grid”, “tidyverse” and “gcookbook” R packages were used for analyzing data and illustrating results.

## Results

### Variable correlations

**Fig 2:**
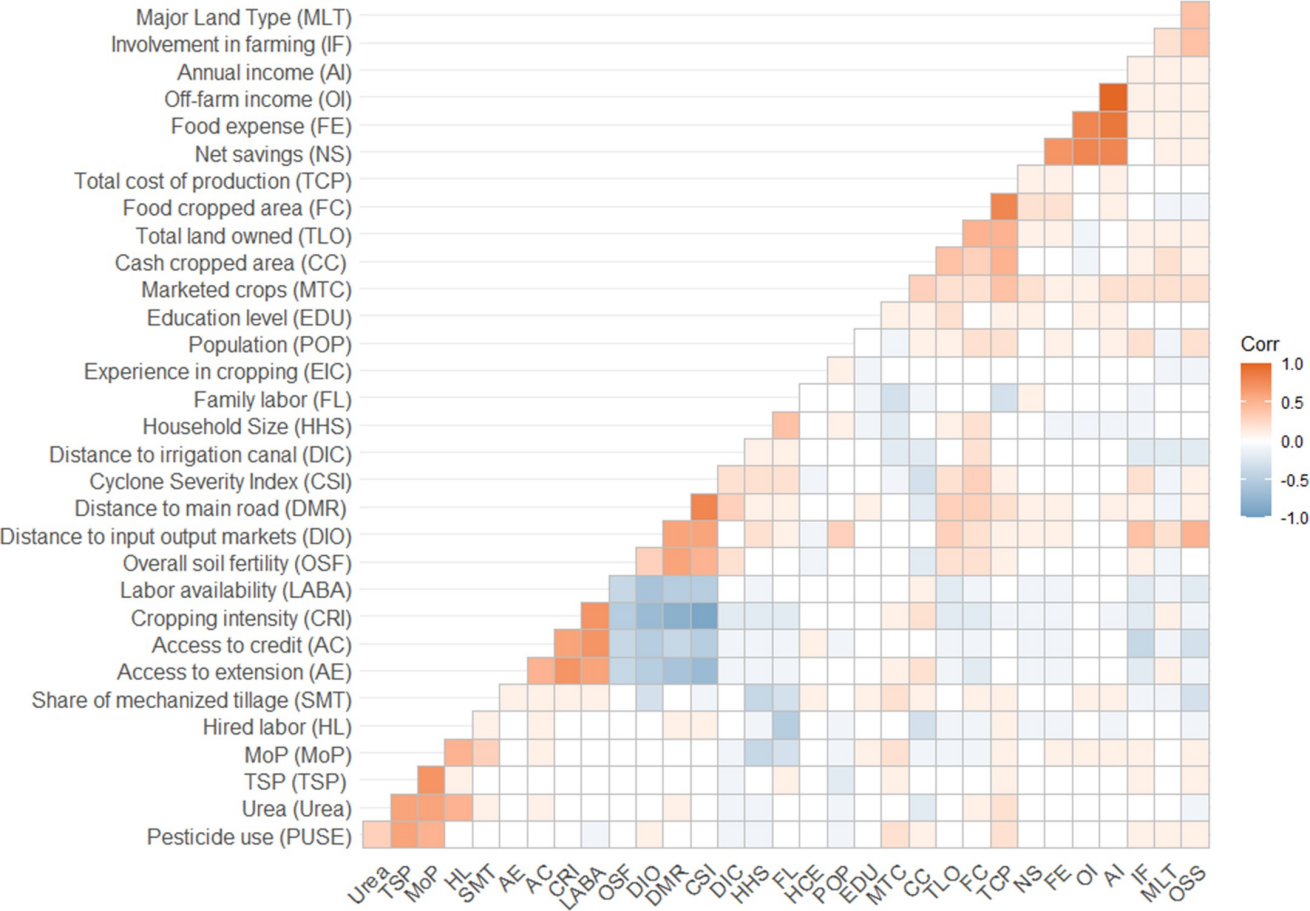
Correlation (corr) matrix of variables collected through smallholder household survey.

The correlation matrix showed that household variables in the survey dataset were largely independent (Fig 2). In several cases, high correlations occurred when variables belonged to the same group. For example, households that applied fertilizer inputs also applied pesticides, so these variables were positively correlated with each other. Economic variables were also positively correlated (off-farm income, food expenses, and annual net savings). The group of variables consisting of cropping intensity, labor availability, and access to credit and extension showed the strongest negative correlation with the group of variables including overall soil fertility, field distance from main road, distance to input-output markets, and cyclone severity index. However, within these groups, there were some positive relationships among variables. For example, labor availability and access to extension and credit positively influenced cropping intensity.

### Composite variables produced through factor analysis

Using these 32 variables, factor analysis identified five distinct factors that represented 51% of variance in the dataset (Table 2). These five factors were uncorrelated, meeting an important assumption of this method (S1 Fig). Higher loading scores in Table 2 represent more influential variables (shown in bold), which can have positive or negative relationships with each factor. For the purposes of this study, each factor was assigned a label considering the most important variables contributing to it—either the top five variables or those with >0.40 loading scores (Table 2).

**Table 2:** Factors representing key differences in rice-based smallholder farming systems of coastal Bangladesh.

Variables	Factor 1	Factor 2	Factor 3	Factor 4	Factor 5
	Farming Challenges	Economic Status	Crop Management Practices	Asset Endowment	Farm Characteristics
Major Land Type (MLT)	-0.07	0.06	0.00	0.04	**0.44**
Household head involvement (IF)	0.19	0.05	0.09	0.07	**0.49**
Household annual income (AI)	0.03	**1.00**	0.03	0.07	0.00
Household Off-farm income (OI)	-0.01	**0.99**	0.07	-0.08	0.01
Household food expense (FE)	0.05	**0.86**	0.01	0.08	0.05
Household annual net savings (NS)	0.06	**0.81**	-0.02	0.12	0.03
Household total cost of production (TCP)	0.07	0.02	0.21	**0.93**	-0.05
Food cropped area (FC)	0.27	0.08	-0.05	**0.84**	-0.24
Total land owned (TLO)	0.21	-0.01	-0.05	**0.55**	0.11
Cash cropped area (CC)	-0.30	-0.04	-0.16	**0.65**	0.29
Percent produced crop marketed (MTC)	-0.14	0.16	0.24	0.39	0.24
Farmer education level (EDU)	0.00	0.05	0.09	0.09	-0.03
Village population (POP)	0.04	0.05	-0.22	0.22	0.12
Households’ cropping experience (HCE)	-0.05	0.03	0.01	0.00	-0.08
Family labor per ha (FL)	0.17	0.01	-0.35	-0.21	0.00
Household Size (HHS)	0.21	-0.11	-0.37	0.05	0.00
Distance to the irrigation canal (DIC)	0.26	0.01	-0.13	0.01	**-0.42**
Cyclone Severity Index (CSI)	**0.87**	0.01	-0.04	0.01	-0.06
Distance to the main road (DMR)	**0.85**	0.02	0.06	0.11	-0.10
Distance to the input-output market (DIO)	**0.70**	0.01	-0.09	0.13	**0.46**
Overall soil fertility (OSF)	0.59	0.01	0.00	0.08	-0.12
Labor availability (LABA)	**-0.70**	-0.03	-0.01	-0.01	-0.27
Cropping intensity (CRI)	**-0.94**	-0.03	0.03	0.00	0.00
Access to credit (AC)	-0.62	-0.04	0.10	0.01	-0.34
Access to extension (AE)	**-0.74**	-0.03	0.01	0.02	-0.04
Share of mechanized tillage (SMT)	-0.15	0.08	0.29	0.14	-0.27
Hired labor per ha (HL)	0.10	-0.09	**0.58**	-0.06	-0.18
Muriate of potash rate (MoP)	-0.01	0.06	**0.86**	-0.06	0.10
TSP rate (TSP)	0.03	-0.02	**0.67**	-0.01	0.23
Urea rate (Urea)"	0.10	-0.01	**0.76**	0.01	-0.11
Pesticide use (PUSE)	0.01	0.02	**0.50**	0.10	0.24
Overall soil salinity (OSS)	0.12	0.06	-0.04	0.04	**0.70**
SS loading	5.10	3.44	2.96	2.67	2.02
Proportion of Variance	0.16	0.11	0.09	0.08	0.06
Cumulative Variance	0.16	0.27	0.36	0.44	0.51
Proportion of Variance explained	0.31	0.21	0.18	0.17	0.12

Higher loading scores represent more influential variables for each varimax-rotated factor (shown in bold). Factors were named based on the top variables contributing to each factor.

Overall MSA = 0.72; RMS off-diagonal residuals = 0.056.

Factor 1 was named ‘*Farming challenges*’ because it represents household crop production challenges, where cropping intensity and access to extension had a negative influence, and cropping intensity, distance from main road, and distance to input and output markets had a positive influence on the factor score. Factor 2 was termed ‘*Economic status*’ due to the positive loading of off-farm income, annual income, food expense, and net savings, all representing positive impacts on farm household livelihood. Factor 3 was titled ‘*Crop Management Practices*’ because of the strong positive influence of fertilizer use, pesticide inputs, and hired labor on factor scores. Factor 4 was named ‘*Asset Endowment*’ due to the positive loading of total land owned, food cropped area, cash cropped area, and total cost of production. Factor 5 was named ‘*Farm Characteristics*’ because of the strong positive influence of overall soil salinity, distance to the input-output markets, major land type, and household heads’ involvement in farming. While *Farming Challenges* accounted for 31% of explained variation in the dataset, the factors *Economic Status*, *Crop Management Practices*, and *Asset Endowment* also each accounted for 17–21% of the explained variation among households, whereas Farm characteristic accounted for 12% of variation.

### Changes over time

The multi-level model results indicate that only *Crop Management Practices* was significantly affected by Year (Table 3). All factors except *Farming Challenges* and *Economic Status* were affected by Embankment status (farms located within vs. outside of polders). The three factors, *Farm characteristics*, *Asset endowment*, and *Farming challenges*, were affected by a Year by Embankment status interaction.

**Table 3:** Multi-level model summary showing the effects of year and embankment status on each factor (latent variable).

Effect	Composite variables (*P*-value)				
	Farming Challenges	Economic Status	Crop Management Practices	Asset Endowment	Farm Characteristics
Year (Y)	NS (0.76)	NS (0.07)	<0.001***	NS (0.99)	NS (0.66)
Embankment status (ES)	NS (0.84)	NS (0.19)	0.02*	0.03*	<0.01**
Y × ES	<0.001***	NS (0.36)	NS (0.13)	<0.001***	<0.001***

In the best-fit model, Year and Embankment status were designated as random and fixed effects, respectively. Smallholder households were nested within Embankment status (polder vs non-polder).

For random effects P(>Chisq) and for fixed effects P(>F).

Significant code ‘***’ 0.001 ‘**’ 0.01 ‘*’ 0.05.

**Fig 3:**
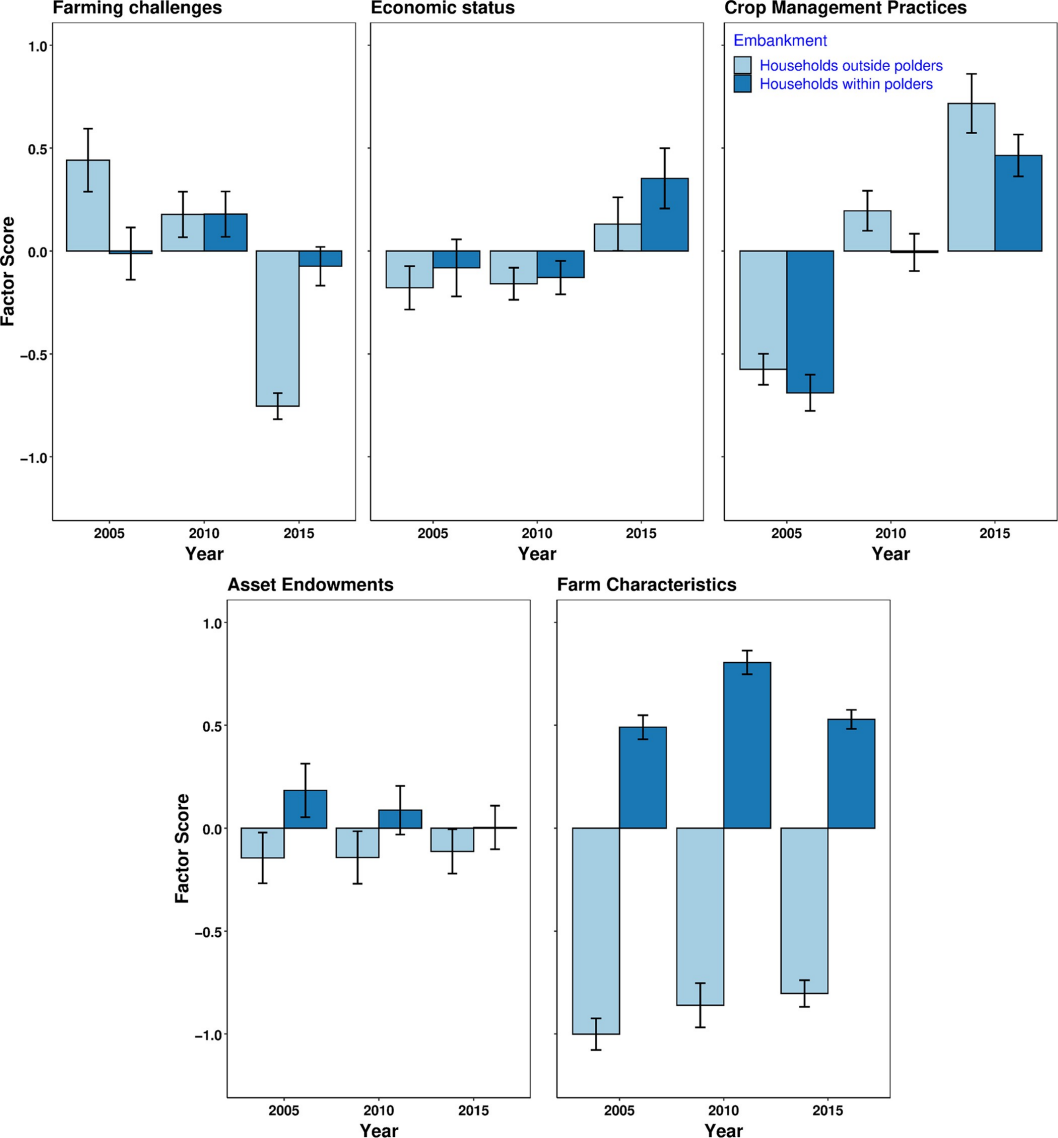
Change in factors over time during the study period (error bars indicate 95% confidence intervals). Factors are independent and represent key differences in smallholder farming systems for households within and outside polders in the study area.

**Fig 4:**
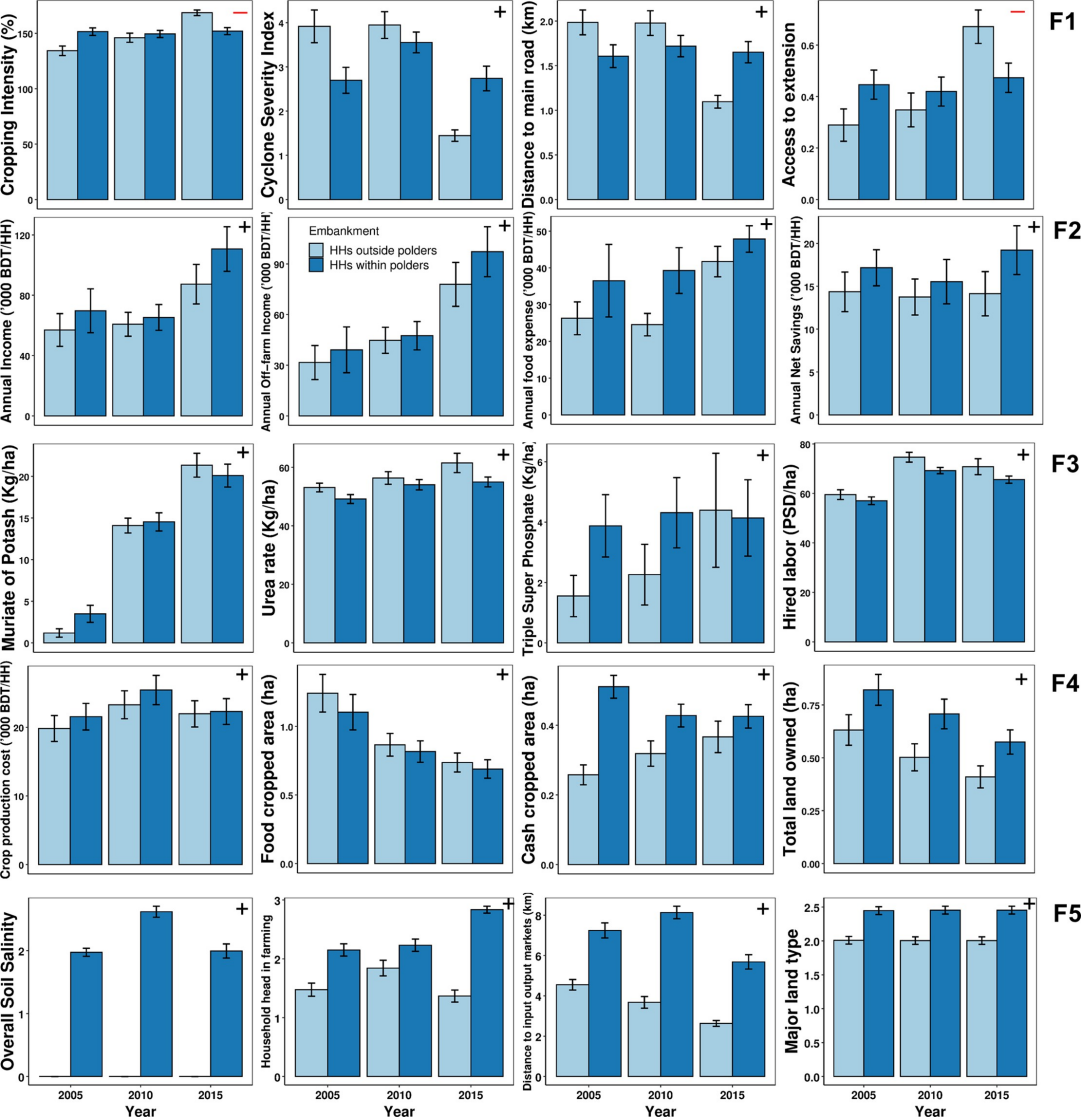
Changes over time in most influential variables contributing to each latent factor during the study period (2005–2015) (error bars indicate 95% confidence intervals). The associated factor from Fig 3 is displayed in each panel. (F1 = Farming challenges, F2 = Economic status, F3 = Crop Management practices, F4 = Asset endowment, F5 = Farm characteristics, and ‘+’ = Positive loading, ‘-’ = Negative loading, HH = Household).

Model predictions (mean ± 95% confidence intervals) from the ANOVA are displayed in Fig 3. The factor *Farming Challenges* decreased over time in households outside polders from 2005 to 2015, whereas it was more variable for households within polders. High loading scores for the most important variables showed that increased cropping intensity, better access to extension, decreased distance to both the main road and input-output markets, and decreasing cyclone severity index with time contributed to a decrease in *Farming challenges* in non-polder households, whereas trends for these variables for polder households were more variable and most of them did not improve over time (Table 3 and top row of panels in Fig 4). For example, for within polder households, except distance to input-output markets that decreased with time, other variables (cropping intensity, cyclone severity index, and distance to main road) did not improve from 2005 to 2015. Changes in the factor *Economic status* (Significant at 90% level) in both polder and non-polder households (Fig 3; Table 3) were associated with an increase in annual income, specifically from off-farm activities, corresponding with an increase in annual net savings (second row of panels in Fig 4).

The factor *Crop Management practices* increased over time for households both within and outside polders (Fig 3). The increase was relatively greater in non-polder than polder households. Important variables owing to this change included higher NPK fertilizer inputs, with muriate of potash (MoP) showing the highest loading score, for which application rates considerably increased over time (Table 2, second row of panels in Fig 4). Urea application also increased over time (third row of panels in Fig 4). Triple superphosphate fertilizer increased over time in outside polder households but did not change within polder households. Two other variables contributing to an increase in *Crop Management Practices* were increasing hired labor used for crop production and pesticide use. The use of these variables in crop production also increased from 2005 to 2015.

Changes in *Asset endowment* were less noticeable, with a decreasing trend observed only for households within polders. The total amount of land owned by these households decreased over time; this was associated with a corresponding decrease in area devoted to cash crops and food cropped area (bottom row of panels in Fig 4). At the same time, these households faced greater total costs of production during the middle of the study period. This trend was different for households located outside polders, which increased cash cropped area over time. These households, however, also reduced food cropped area, similar to households within polders. Finally, *Farm characteristics* showed distinct differences between households within and outside embankments (Fig 3). Differences in this factor were primarily related to overall soil salinity levels (Table 3), with high soil salinity observed for households within polders due to their relatively closer proximity to the coastline (bottom panel in Fig 4). In addition, decreasing distance to canals that could be used for surface water irrigation and changes in the involvement of the household head in farming were influential in households outside polders (Fig 4).

### Crop productivity

Crop productivity increased by around 22% in 2015 compared to 2005 across both households within and outside polders (S2 Fig). Of the different crops grown in these systems, *aman* rice contributed more than 60% to household annual crop production. All households practiced *aman* rice followed by fallowing on some portion of their land, with many also cultivating mung bean and lathyrus as a second crop. Mung bean replaced lathyrus over time as the second largest contributor to household annual crop production, reaching approximately 20% by 2015.

The influence of the latent factors on crop production depended the scale of REY considered. At the household level, which reflected total annual crop production across all cropped area, *Asset Endowment* followed by *Farming Challenges* and *Farm Characteristics* were ranked as the most important factors influencing total household annual crop production (S4 Fig). However, when crop productivity was expressed on a per unit area basis, the most important factors were *Crop Management Practices* followed by *Farm Characteristics* and *Economic Status* (S4 Fig). The factors *Crop Management Practices* and *Economic Status* had a significant positive effect on household crop productivity (t ha^-1^), while *Farming Challenges* had a negative effect, and *Farm characteristics* for non-polder households had a mixed response.

### Pathways out of poverty

The household poverty trajectory map illustrates the transition of households between different categories of per capita per day income between 2005 and 2015 (Fig 6). In 2005, 97% of households lived below the poverty line, with income less than one dollar per capita per day (categories C and D). By 2015, however, 21.5% of households were above this threshold (≥ one dollar per capita per day, categories A and B). Results also showed that as the proportion of off-farm income to total annual income of the household increased, households with per capita income < one dollar per capita per day decreased (S1 Table). Importantly, many households moved out of poverty from categories C and D during the study period, with 41% of households originally in category C moving into categories A or B, and around 18% of households originally in category D moving into categories A or B. Across all groups, households moved out of poverty by increasing their off-farm income, which replaced field crop production as the dominant contributor to total household annual income. For example, in 2005, income from field crop production and off-farm activities represented 72.2% and 27.8%, respectively, of annual income. But by 2015, the contribution of off-farm income increased to 74.4% of annual income, while the contribution of field crop income decreased to 25.6% (S1 Table).

## Discussion

Across a comprehensive set of 32 variables influencing food security and rural livelihoods, our results indicate that critical improvements have occurred in rice-based smallholder farming systems in coastal Bangladesh during the study period. Considering the five factors developed in this study, households in Bangladesh’s central coast were better off in 2015 than in 2005, both within and outside of polder systems, particularly for the variables contributing to the factors *Crop Management Practices* and *Economic Status*. These positive results are consistent with recent reports for southern Bangladesh, where on average, farmers have improved food security and economic status [29]. This has occurred despite significant changes to smallholder farming systems in this region. Using the same panel dataset to assess drivers causing the differentiation of farm typologies, Aravindakshan et al. (2020) found an increase in smaller and more marginal farms due to the subdivision of land and plot fragmentation, combined with increasing homogeneity of agricultural systems [15]. However, these negative outcomes were offset by increased off-farm activities, leading to greater annual income for vulnerable households.

A key challenge in agricultural development–particularly in the tropics and sub-tropics–is understanding the diversity of smallholder systems and major constraints limiting crop productivity, among a wide range of potential challenges [2]. The contribution of our study was to first develop latent factors that explained variation among households and then assess the relative impacts of these factors on crop productivity, providing new insights into policy options for agricultural and environmental development in Southern Bangladesh. Our premise is that by evaluating the variables contributing to latent factors, it is possible to understand the specific function of that factor in the trajectories of development in smallholder systems. In turn, the role of each factor can be improved by creating targeted government programs and policies to address variables with the highest loading scores, in addition to variables that are highly correlated with those variables.

### Overcoming *Farming Challenges*

Among the range of variables studied here, *Farming Challenges* explained the highest proportion of variance in our factor analysis (31%). This finding is important for designing strategies to best support smallholders in a disaster-prone tropical coastal zone like southern Bangladesh. It is well-documented that the top three variables contributing to this factor (cyclone severity index, distance from farms to main roads, and distance to input-output markets) are major issues influencing farmer livelihoods in coastal South Asia [21, 39, 60–62]. For example, the damage from Category IV cyclone Sidr totaled $1.67 billion, including damage to 0.6 million ha of cropland, with 8.9 million people affected in southern Bangladesh in 2007 [63]. The negative impact of tropical cyclones is a recurring issue, and this trend is anticipated to increase with climate change and rising sea surface temperatures in the Bay of Bengal [5]. Resulting crop damage can be particularly devastating because tropical cyclones are most frequently hit during both planting and harvesting times in April, May, October, November, and December [5]. While all variables studied here might affect the performance of smallholder systems to some degree, this finding highlights the need for effective strategies to decrease the vulnerability of smallholders to these climate shocks. Two main approaches to achieve this goal include building or improving roads and embankments to protect against flooding and saltwater intrusion during storm surges [39, 64], and helping smallholders develop resilience through climate change adaptation strategies. Farmers in Bangladesh’s broader coastal region currently use multiple adaptation strategies, including selective reduction of household food consumption, off-farm employment, seeking assistance from government and NGOs, and using savings or borrowing funds from family and neighbors to deal with climate risks [65]. Both of these vulnerability decreasing strategies could be further strengthened with sufficient research and policy support.

We observed that better access to roads and input-output markets was critical for smallholders to respond effectively to farming challenges. Well-planned infrastructural development (both roads and markets) can enhance agricultural and economic growth [22]. Rural road development in Bangladesh has been shown to decrease poverty by facilitating favorable conditions to increase agricultural production and expand market accessibility. The latter can assist in reducing input and transportation costs incurred by farmers [21]. Road improvements can also facilitate access to governmental and non-governmental services, including extension, credit, health, and education, among others. However, because construction results in the formation of depressions resulting from the excavation of soils to build ridges upon which roads are placed, it should be considered that constructing new roads may also increase waterlogging [66] and reduce cropland area, which can put other pressures on agricultural productivity. As discussed below, food cropped area was a strong contributor to the factor *Asset Endowment*, in addition to total costs of production and land ownership. Because this factor most strongly influenced annual household food production, we suggest that government efforts to benefit smallholder livelihoods through road development should also consider, and hedge against, these potential trade-offs in agricultural productivity.

In our dataset, better access to roads and markets was also associated with the next three most important variables contributing to a reduction in *Farming Challenges* during the study period—increased cropping intensity, as well as better access to extension and labor availability (Fig 2). The observed cropping intensity (average 150% in 2015) in the study area is low compared to the national average of 190% [36]. Research in the coastal region has documented several causes for low cropping intensity, particularly poor extension services, tidal flooding, poor access to input-output markets, soil salinity, and inadequate use of water resources for irrigation [16, 67, 68]. Poor soil fertility may also discourage smallholders from producing crops with higher costs of production, particularly dry *rabi* season crops, which limits the number of crops grown per year. Cropping intensity also varies widely depending on inundation land types, with higher cropping intensity found on land that is inundated less often [67]. However, most of the land in southern Bangladesh is medium highland that experiences 30 to 90 cm flooding depth during monsoon, suitable for two to three crops per year [67, 68]. While many of the challenges described above are present in the study region, the experience of our households survey suggests it is indeed possible to increase cropping intensity, with at least 12% of households having a cropping intensity over 190%. Households largely achieved this by increasing the cultivation of fallow lands during the dry season, which has also been documented as a significant opportunity through research [16].

Reinforcing our findings, agricultural extension plays a vital role to collaboratively develop and disseminate improved technologies and knowledge to and among smallholders [62]. Extension services can have positive influence on increasing cropped area, economic participation in the household, and partial or full entry into commercial agriculture [60]. However, studies have observed poor access to and limited support for appropriate extension services in Bangladesh [25]. Another example is that approximately 60% of farmers rated available services as low quality for both NGOs and public extension services [69]. To improve extension services, emphasis should be placed on not only expanding programs and access but also on enhancing the quality of extension. This requires sufficient funding to ensure that well-trained extension workers have resources for program development and participatory farmer engagement and also new research to develop more effective tools and disseminate knowledge and technology to smallholders in remote coastal areas. In particular, the Department of Agricultural Extension (DAE) in Bangladesh has no research wing, and scientifically sound studies on extension methodologies are relatively rare among national agricultural research institutes. A key structural change that could address this challenge is the development and inclusion of extension effectiveness research alongside existing project-based monitoring and evaluation services within DAE.

To support a transformation of smallholder systems towards increased cropping intensity, it is important to consider other strongly correlated variables in our dataset, particularly access to extension and labor availability, while reducing the distance to roads and markets, and cyclone severity (Fig 2). The significant interaction for *Farming Challenges* depending on household embankment status helps illustrate this example, where this factor decreased over time in households outside but not within polders, mostly by 2015 (Table 3; Fig 4). In non-polder areas, distance to main roads and markets decreased during the study period, likely due to new road construction, thereby facilitating improved access to extension and credit, which can ultimately help to increase cropping intensity, a key factor influencing household annual crop production. In contrast, most of these variables remained stable in polder areas. These results suggest that polder regions require more attention to address the issues related to *Farming Challenges* and *Farm Characteristics* to sustainably intensify rice-based cropping systems and improve smallholder livelihoods.

### Intensification of crop production

**Fig 5:**
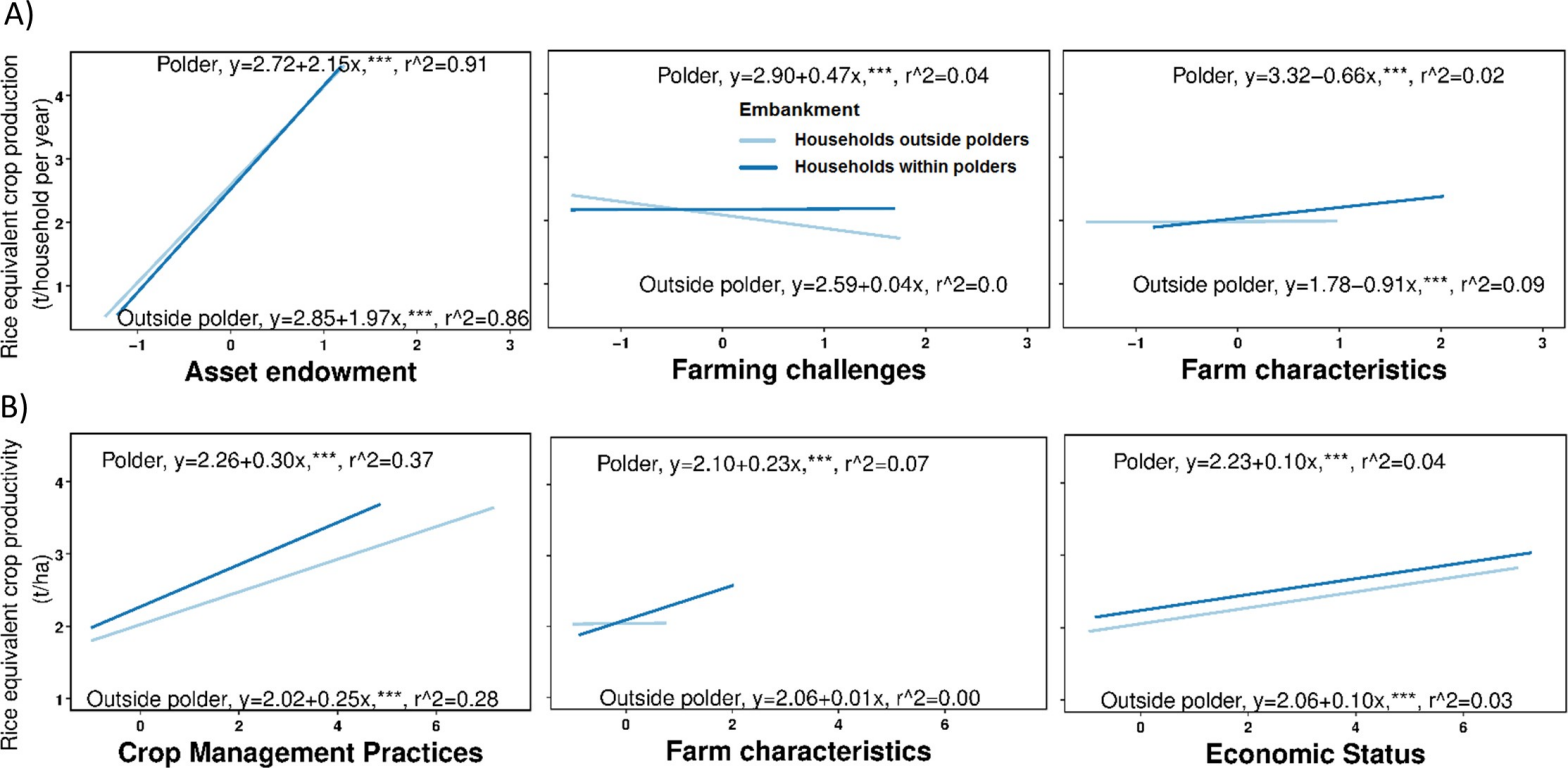
Relationship between influential factors and farm-level crop productivity for households within and outside polders (at 95% confidence level). A) Model predictions showing the effect of the three most important factors on household total annual crop production in terms of rice equivalent yield (REY), B) Model predictions showing the effect of the three most important factors on household crop productivity per unit area (REY per ha).

The factor *Asset Endowment* had the greatest impact on household annual crop production, particularly through decreases in food cropped area and total land owned in both polder and non-polder systems. As a result, household annual crop production was found to decrease over time with declining *Asset Endowment*, which was significant for households within polders (Fig 3, Fig 5A, S2 Fig). Despite the importance of maintaining or increasing food cropped area in our analysis, current trends in Bangladesh suggest that overcoming this challenge will be difficult. Per capita arable land is around 0.05 ha and is decreasing over time [70]. Similarly, we observed that an increase in smaller and more marginal farms was associated with declining household annual crop production during the study period (Fig 5; S2 Fig). Limited access to credit and savings presents additional challenges to increasing farm size because an important consequence of farming in more areas is higher total costs of production. However, limited access to arable land as the primary constraint to increasing annual crop production can be approached from a different angle, that is, through heightened cropping intensity. Experts in Bangladesh have promoted closing yields gaps as a primary path towards food security, emphasizing the opportunity to optimize crop productivity through changes in temporal, in addition to spatial, management of land resources [35]. Based on our findings, efforts to increase cropping intensity should be complemented by improved management practices (next section) to increase crop productivity, both on a per unit area and per household basis. For example, one recent study observed 43% to 64% higher crop production due to increasing cropping intensity compared to only improving crop management within current systems in Bangladesh [71]. Bhattacharya et al. (2019) demonstrated that 14–20 t ha^-1^ REY can be achieved with cropping system intensification in the polders of low salinity coastal zones of Bangladesh, with the potential to achieve a gross margin of USD 1200 ha^-1^ yr^-1^ [72].

In our study, *Crop Management* had the greatest impact on crop productivity per unit area, largely owing to increased external inputs. As found in smallholder systems elsewhere [73], the large response of crop productivity to nutrient inputs can be explained through baseline soil fertility levels and fertilizer use. Soil fertility in coastal Bangladesh is generally low, with soil pH varying from 5.0–8.0 combined with low soil organic matter (0.1–2.3%), total N (0.1–0.3%), P (4–28 ppm), and K (0.2–0.7 m.e.%) (Haque, 2006). The low soil fertility of coastal farms enhances the dependency of crop productivity on fertilizers, pesticides, and labor for management (Table 1; Fig 2; Fig 5; Devkota et al., 2019; Haque, 2006). To boost farm-level productivity, the government of Bangladesh started subsidizing imported urea, triple superphosphate, and muriate of potash from 2005 [48]. This might have triggered the increased use of fertilizers in our study region, particularly MoP and TSP (Fig 4), which strongly contributed to the factor *Crop Management Practices* and ultimately enhanced crop productivity.

Though we observed an increasing (22%) trend in crop productivity, our study area remains one of the lowest productivity zones nationally [14], highlighting the urgency of increasing yields of the major crops grown including rice, mung bean, grass pea, vegetables, and other dry season crops. Our findings suggest one reason for low rice productivity is poor fertilizer management. Recommended rates (kg ha^-1^) of NPK for the transplanted *aman* crop with a local variety (yield target 3.0±0.30 t ha^-1^) and an improved variety (yield target 5.0±0.50 t ha^-1^) are 45-6-10 and 75-10-18, respectively, for saline areas within polders, and 75-10-18 outside polders in non-saline areas generally [74]. However, on average, smallholders in our study applied a 31.5–0.0–14 rate for *aman* rice in 2015, much lower than the recommended rate [24]. This could be a result of inadequate fertilizer and pesticide supply through market channels or higher prices of these inputs during the cultivation season [14]. In line with this result, household economic status also positively influenced crop productivity in our data (Fig 5B). Similarly, access to credit was another variable associated with overcoming this dimension of *Farming Challenges* (loading -0.62, Table 1), indicating that economic status and access to credit have benefits corresponding to higher production. It has been estimated that a 10% increase in agricultural credit may positively influence productivity at 1.2 t ha^-1^ or 8.5% [75, 76]. The lack of easy access to credit and limited understanding of financial institutions and high interest rates are cited as the main reasons for farmers not using credit [14], which further underscores the need for strong and multi-faceted extension services discussed above.

The third factor influencing crop productivity per unit area was *Farm Characteristics*, which was composed of the variables access to irrigation and soil salinity. In the overall factor analysis, this factor showed some of the greatest differences for households within versus outside polders, largely due to greater salinity problems for the former households, which are closer to the coast and susceptible to seawater intrusion. Although we did not observe a large impact of this factor on crop productivity in polder areas, this result could be misleading and requires further interpretation. This variable had little influence because the current farming system is dominated by rainfed transplanted aman and non-irrigated pulses. Yet at the same time, lack of irrigation is one of the main causes of the current low productivity of rainfed crops and also a barrier to farmers’ diversification into other crop species that benefit from irrigation.

As noted above, access to irrigation and drainage is therefore an important aspect to sustainably increase cropping intensity and diversified crop production by cultivating dry season fallow land in south-central Bangladesh [16]. Similarly, farmers have adapted their crop calendar to avoid soil salinity effects which are most extreme from March through May [77, 78]; however, this transition may result in low productivity. The yield penalty of growing crops beyond the optimum planting date is well-established in southern Bangladesh [72, 78], although this effect can be partially avoided by adapting saline tolerant varieties/crops. Based on previous studies in south-western Bangladesh, we may also interpret that lower tidal flooding in polder areas might be the reason for polders’ positive impact on increasing crop productivity and annual crop production, while no effects were observed in non-polder areas (Fig 5; Adnan et al., 2019; Haque et al., 2018; Zaman and Mannaf, 2016; Chowdhury et al., 2010).

### Off-farm income as a pathway out of poverty

Our results align with a growing number of studies indicating that off-farm income is key to improving the economic status and food security in smallholder systems [33, 77, 79]. Many smallholders are unable to invest in agricultural best management practices and technologies due to their poor economic status; hence they are often net buyers of crops [80], further contributing to food insecurity and poverty. Where crop production has become less profitable, farmers in sub-Saharan Africa and Asia are widely seeking opportunities for off-farm income generation [32, 33], in order to feed their families and invest in other non-agricultural activities.

**Fig 6:**
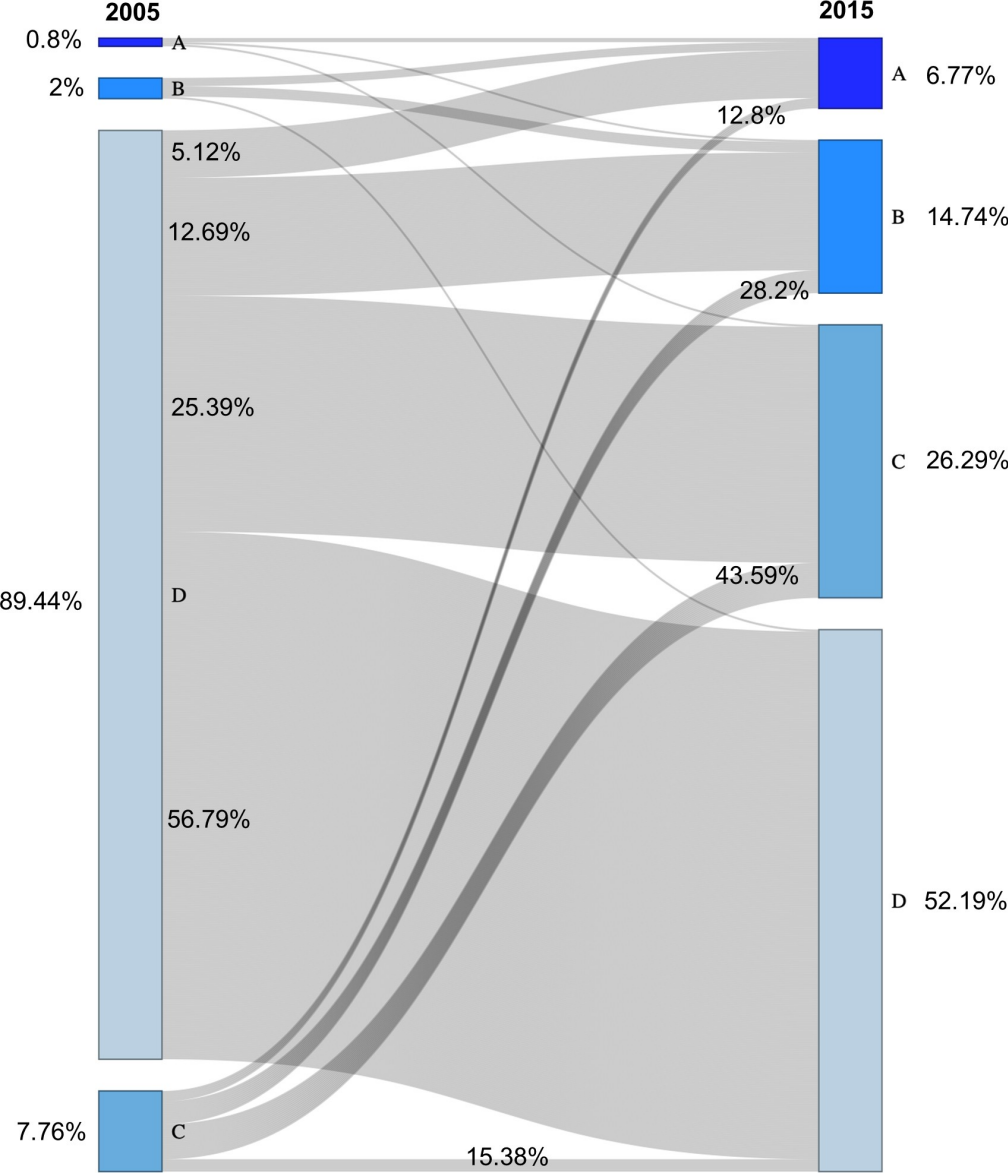
Household poverty trajectory during the study period. Diagram illustrates the movement of households into different categories of income. Note, categories refer to Per capita income per day in USD, A = ≥ 2, B = 1 to 1.99, C = 0.5 to 0.99, and D = < 0.5.

We found that most households in our study have taken similar strategies to improve their economic status, with the household poverty trajectory map highlighting the proportion of smallholders who have moved into higher categories of economic earning (Fig 6). Most importantly, households from any level of poverty had the ability to move out of poverty by increasing their share of non-crop income. For example, to reach the per capita earnings of one dollar USD per day (Category B), which was the established poverty threshold in 2005, households had to increase the proportion of their off-farm income to 81 or 93% of their annual income in 2005 and 2015, respectively (Fig 6; S1 Table). Even among the poorest farmers in our studied households with less than 0.5 USD per capita per day income in 2005 (Category D), almost 18% broke the poverty line in 2015 by reaching this threshold (93% of annual income from off-farm activities). Similar trends have been observed for smallholders in Nepal [79]. A simulation model study also observed a similar trend for coastal Bangladesh [77].

While creating more off-farm jobs for coastal smallholders may help alleviate poverty, the fact remains that most households are still not self-sufficient in food production. Household food expenses can either be covered by off-farm income or increases in crop production, or both. To fill the current gap where households could, in theory, earn their food expenses through crop production rather than off-farm income, we estimate that smallholders would need to increase their present income from crop production by approximately four times, given their households annual deficit (BDT 34387 and 32237 or USD 462.8 and 493.71 in outside and within polder, respectively, S3 Fig). This is extremely challenging to achieve, but recent studies showed prospects to move closer to this goal by adopting new cropping patterns. Given higher dry season yields, particularly for *boro* rice, it has been estimated that maximizing cropping intensity could double overall crop production and profitability in Bangladesh [35, 45, 81]. Bhattarcharya et al. (2019) demonstrated that annual productivity of 14–20 t ha^-1^ and gross income of USD 1200 ha^-1^ yr^-1^ could be achieved by intensifying the cropping system to 300%, along with improved varieties and best management practices [72].

Nonetheless, cropping system intensification will require additional credit, as discussed earlier. It is possible that additional on-farm income could overcome this problem (if reinvested in farming operations), providing extra credit to intensify cropping systems and therefore improve agricultural productivity. While this may be technically feasible on the right land types, many aspects of this proposal demand further research. Heightened intensification may not be feasible for smallholders due to the significant challenges to increasing cropping intensity discussed above. More importantly, securing off-farm income may represent a more attractive and less complicated opportunity, particularly for younger generations. The increasing popularity of non-agricultural professions has already created a labor crisis in the agricultural sector [14]. These trends suggest the need for policies that explicitly support both farm-level food production and opportunities for off-farm income to achieve the SDGs in this region.

### Recommendations

The findings highlight the importance of off-farm income as a pathway to move smallholders out of poverty. Therefore, there is a need for enabling policies to support both agriculture development and off-farm income opportunities for smallholders to achieve food security and no-poverty goals in the region.

The results highlighted the importance of best-bet agronomic practices (e.g., use of recommended fertilizers and pesticides, appropriate stress-tolerant varieties, access to irrigation and drainage, etc.) for improving the productivity of smallholders in the region. Hence, access to credit for external inputs (e.g., fertilizer), access to information and extension services, infrastructure development (access to irrigation and market, polder, etc.) would be needed to accelerate the adoption of best-bet agronomic practices. Government and development agencies should focus on improving credit access, deploying better extension services, and investing in irrigation and transportation infrastructure.

Our findings also highlighted the importance of the total cropped area in enhancing overall annual crop production. Therefore, government policy should focus on protecting arable land from non-agricultural use. At the same time, government and other agencies should focus on increasing total cropped area through enhancing cropping intensity sustainably. But in order to increase cropping intensity, variables related to the factor farming challenges must be addressed, including several of the recommendations from above. Policies and investments should focus on developing infrastructure (polder, road, market), improving access to extension and credit services, and enhancing labor availability or mechanization in smallholder systems.

### Limitations

Our results suggest that factor analysis with varimax rotation can help determine functional predictor variables for household productivity from a larger number of low inter-correlated diverse variables in complex smallholder farming systems. Importantly, this method may apply to any analysis of farming households with a sufficient number of multidimensional variables (often over 10). However, factor analysis has strengths and limitations [82]. One major limitation in our study is that factor analysis explained only 51% of variability in the data, suggesting that variables not captured in our survey were also contributing to differences among households. In addition, depending on the dataset and the number of variables considered, factor analysis may produce factors that are hard to interpret. Another limitation is that data were obtained through farmer surveys. While this approach is common in agricultural development, results from observational studies should only be interpreted as associations between variables, not causal effects. Moreover, farmers were asked to provide detailed information on 32 independent variables covering diverse dimensions over multiple years, which may challenge the quality and accuracy of responses and contribute to measurement error. See Wollberg et al. (2021) for a thorough review of this issue and the implications for data reliability [83].

## Supporting information

S1 Table Contribution of crop and off-farm income to household annual income for different per capita per person income levels. A = Per capita income per day ≥ 2 dollars, B = Per capita income per day = 1 to 1.99 dollar, C = Per capita income per day = 0.5 to 0.99 dollar, and D = Per capita income per day < 0.5 dollar.
(PDF)

S1 Fig Biplots of factors derived from all independent variables. (TIF)

S2 Fig Changes in crop productivity, household annual crop production, and the contribution of different cultivated crops to household annual crop production (% of rice equivalent yield) within polder and outside polder areas over time. (TIF)

S3 Fig The deficit of crop income to fulfill household food expenses. (1 USD = 69.65 BDT).
(TIF)

S4 Fig The importance of the latent factors on households’ annual rice equivalent crop production (t household^-1^ year^-1^) and rice equivalent crop productivity (t ha^-1^). (TIF)
