# Supplementary material for: Factors contributing to farm-level productivity and household income generation in coastal Bangladesh’s rice-based farming systems
Source: PLoS One. 2021 Sep 10;16(9):e0256694. doi: 10.1371/journal.pone.0256694 (PMC8432825; doi:10.1371/journal.pone.0256694)
Supplement: S1 Table — A = Per capita income per day ≥ 2 dollars, B = Per capita income per day = 1 to 1.99 dollar, C = Per capita income per day = 0.5 to 0.99 dollar, and D = Per capita income per day < 0.5 dollar. (PDF) [file pone.0256694.s001.pdf]

S1 Table. Contribution of crop and off-farm income to household annual income for different per capita per person income levels. A = Per capita income per day ≥ 2 dollars, B = Per capita income per day = 1 to 1.99 dollar, C = Per capita income per day = 0.5 to 0.99 dollar, and D =Per capita income per day < 0.5 dollar.

Per capita income level (USD)	Percent contribution in household annual income			
	2005		2015	
	Crop production	Off-farm	Crop production	Off-farm
≥ 2.00	7.35	92.65	3.25	96.75
1.00 - 1.99	18.17	81.83	6.97	93.03
0.50 - 0.99	37.76	62.24	16.49	83.51
< 0.50	76.96	23.04	38.27	61.73
Overall	72.20	27.80	25.60	74.40
