# Supplementary figures and images for: Factors contributing to farm-level productivity and household income generation in coastal Bangladesh’s rice-based farming systems
Source: PLoS One. 2021 Sep 10;16(9):e0256694. doi: 10.1371/journal.pone.0256694 (PMC8432825; doi:10.1371/journal.pone.0256694)

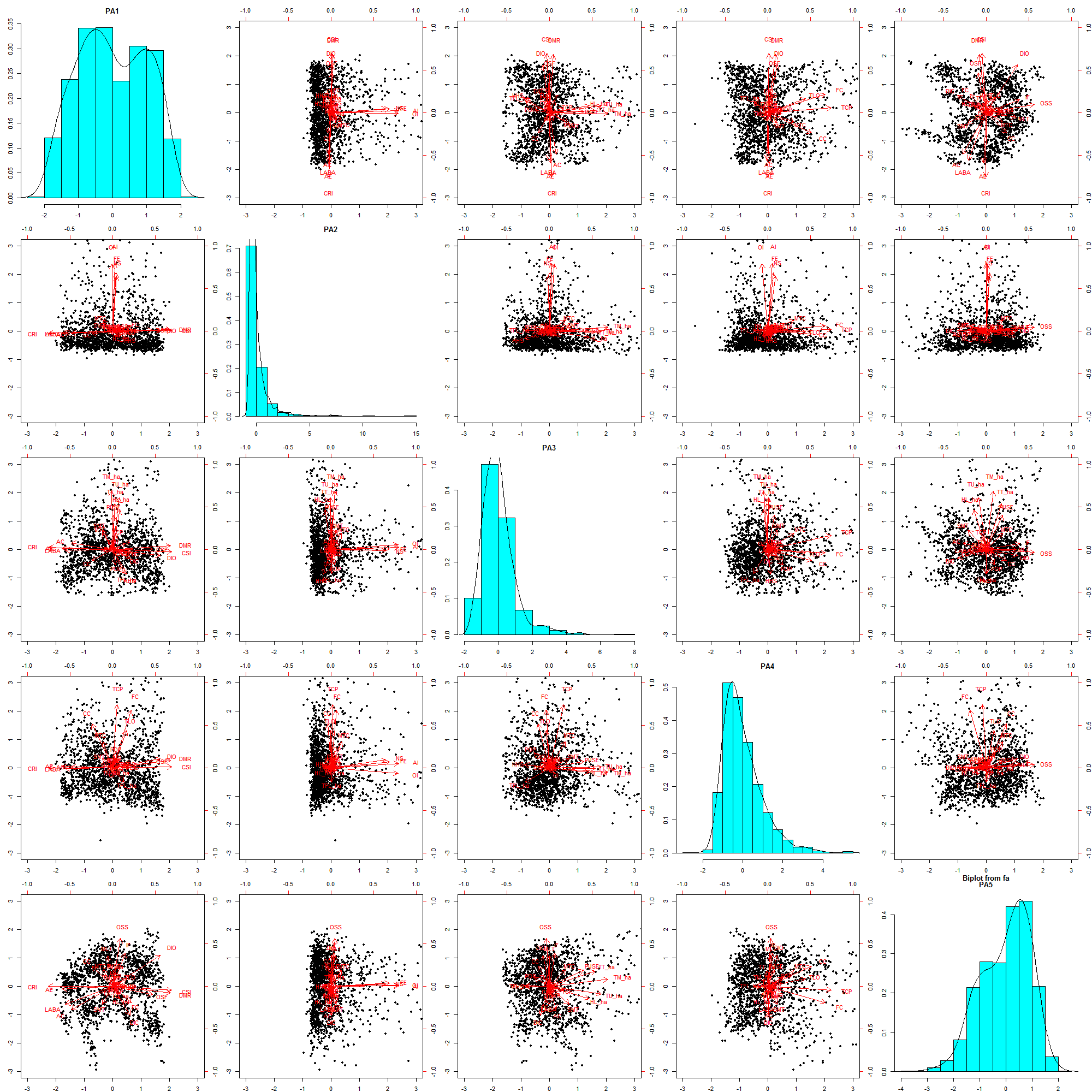

Supplement: S1 Fig — (TIF) [file pone.0256694.s002.tif]

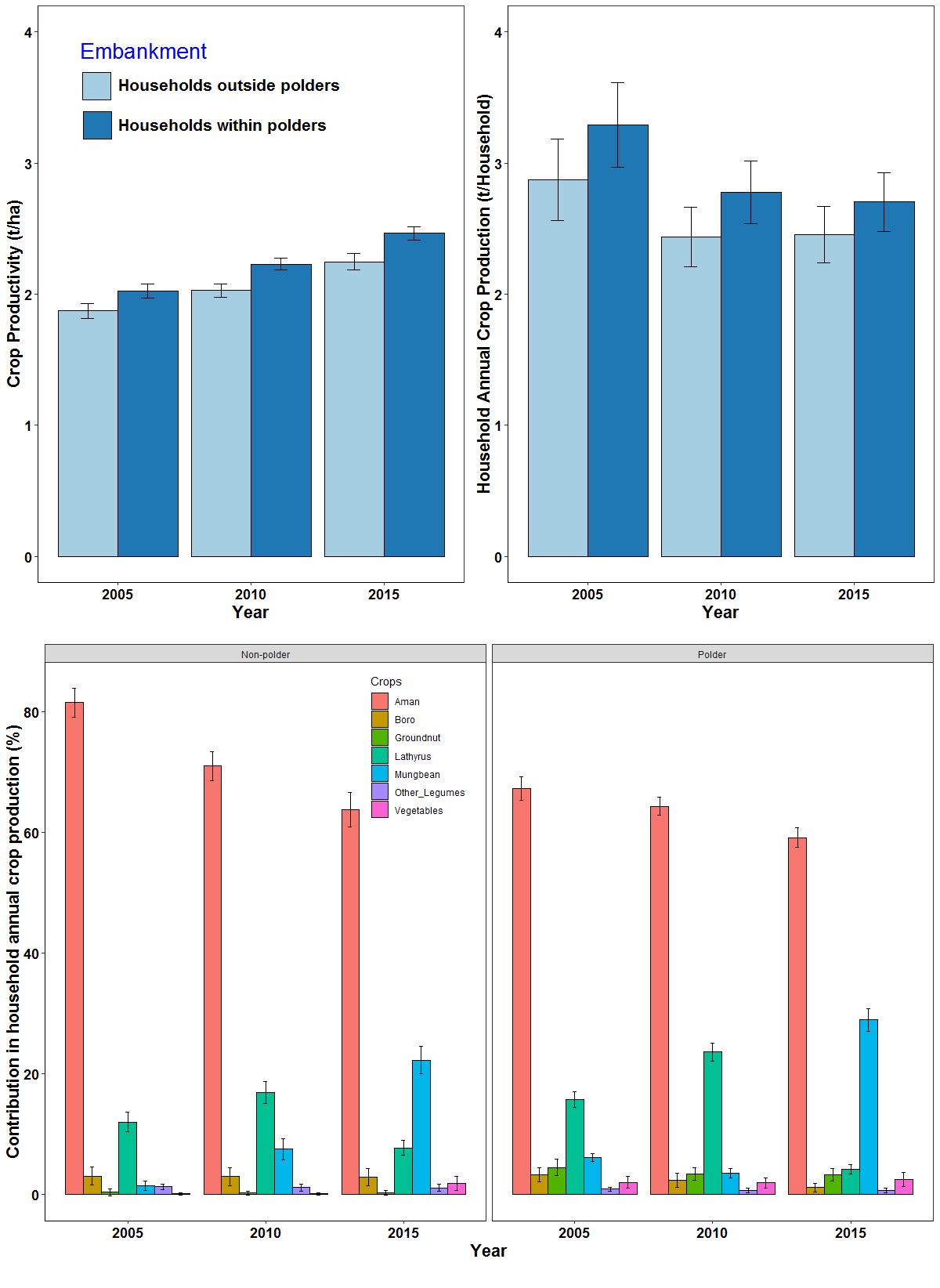

Supplement: S2 Fig — (TIF) [file pone.0256694.s003.tif]

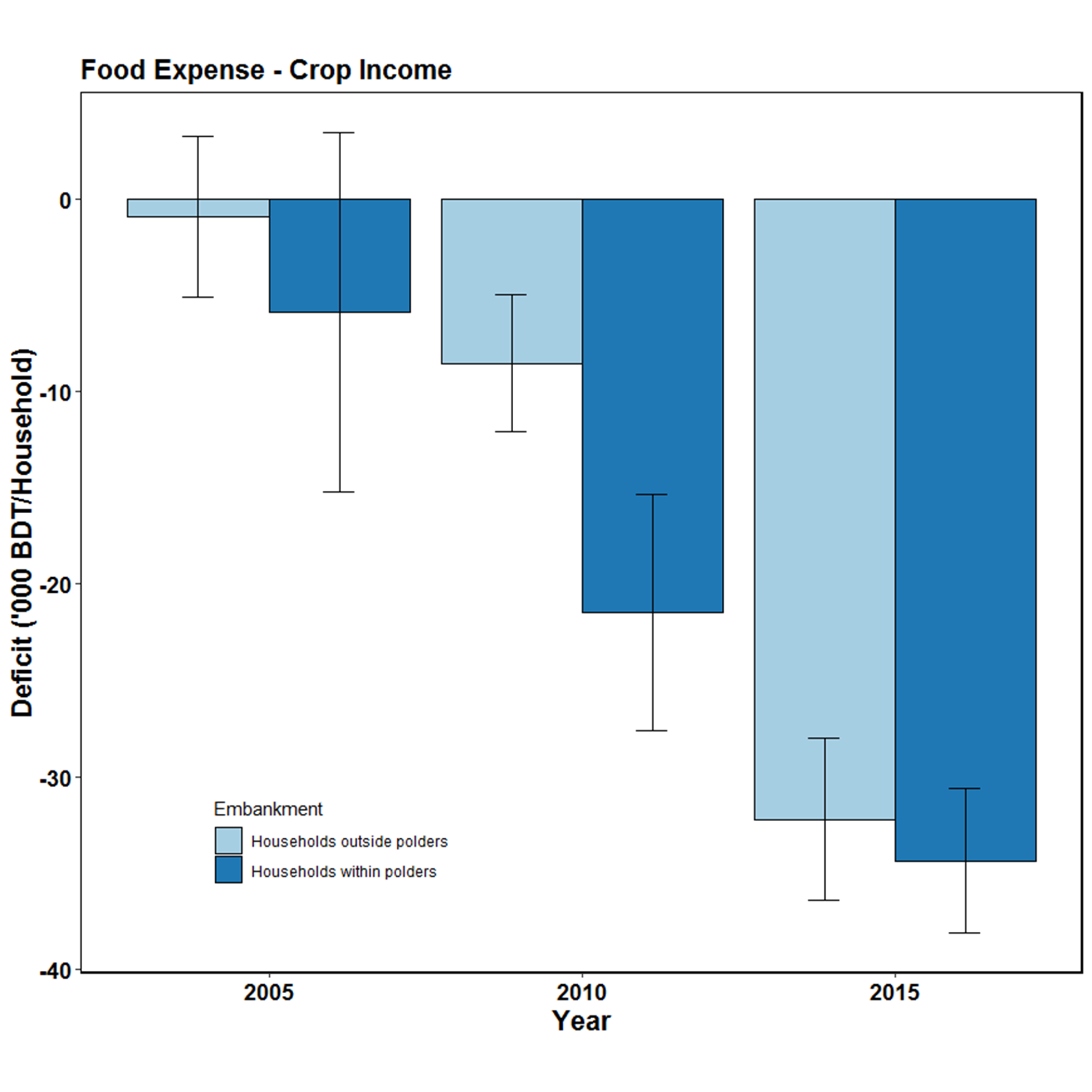

Supplement: S3 Fig — (1 USD = 69.65 BDT). (TIF) [file pone.0256694.s004.tif]

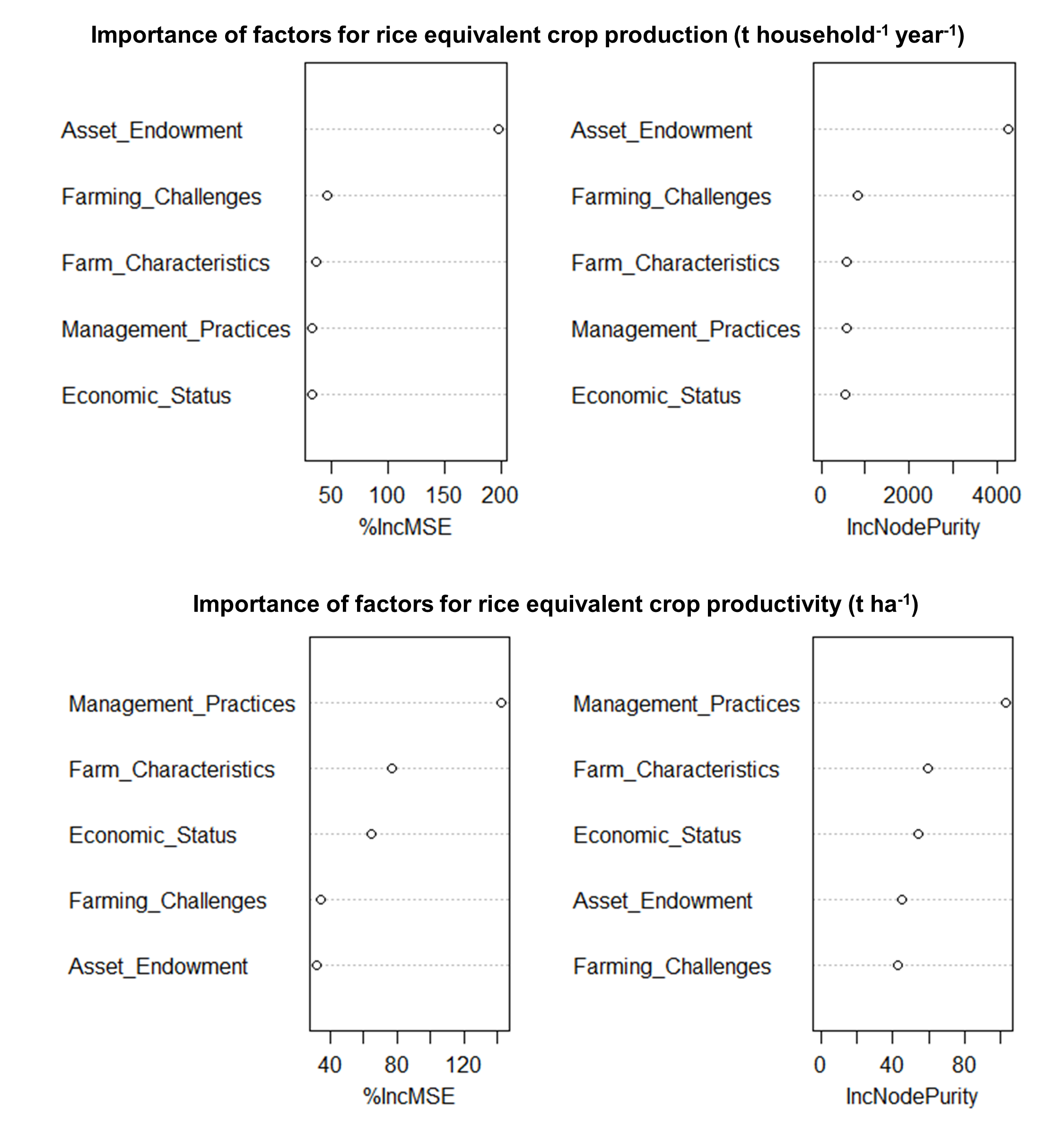

Supplement: S4 Fig — (TIF) [file pone.0256694.s005.tif]
